# Actions of Chloroform and Ether on the Blood Pressure

**Published:** 1914-05

**Authors:** 


ACTIONS OF CHLOROFORM AND ETHER ON THE BLOOD PRESSURE. —H. P. Failrie thus sums up effects of the two anesthetics on blood pressure: A fall of pressure is produced throughout the administration of chloroform. With the establishment of full anesthesia this fall amounts to at least 10 mm. and sometimes considerably more; along with this the pulse beats slower and a varying degree of pallor is present. Surgical shock during chloroform anesthesia produces a slight further fall of pressure. With the withdrawal of these two depressants—chloroform and shock—the blood pressure exhibits a tendency to rise rapidly and very often soon reaches a point a few mm. below the patient's normal. Little alteration of blood pressure is produced by ether. It may cause a rise, it may maintain a constant level, or it may cause a fall. It causes more rapid and more forcible cardiac action, with dilatation of the smaller vessels, as evidenced by flushing; the blood pressure level remains almost constant. With intercurrent shock a considerable fall of pressure takes place, a fall almost equal to the combined effects of chloroform and shock. Recovery from shock is slow, some time elapsing before the blood pressure approaches the normal. Fairlie's summary is based in part on observations made on animals, but mainly on his experiences of three years' use of the drugs in man. It is not generally appreciated that ether thus may lead to an increased danger in the face of surgical shock, and he quotes from several prominent authorities to substantiate this belief. Chloroform would thus seem to be the anesthetic of choice for cases with severe shock, but where shock is absent or likely to be slight, ether should be employed owing to its greater margin of safety.—*Exchange.*

